# Priapism as an Initial Presentation of Sickle Cell Disease: A Case Report

**DOI:** 10.7759/cureus.101553

**Published:** 2026-01-14

**Authors:** Serkalem G Abebe, Constance Cleveland, Maya Kohavi, Saba Zafar, Brandon Yanik

**Affiliations:** 1 Internal Medicine, Henry Ford St. John Hospital, Detroit, USA; 2 Internal Medicine, Wayne State University, Detroit, USA; 3 Internal Medicine, Michigan State University College of Osteopathic Medicine, East Lansing, USA

**Keywords:** exchange transfusion, hb electrophoresis, hydroxyurea use, priapism, sickle cell disease, sickle cell disease screening, urologic emergency

## Abstract

Priapism is a urological emergency defined as an erection lasting more than four hours unrelated to sexual stimulation. Most cases are ischemic and often related to hematologic disorders, especially sickle cell disease (SCD). About 30 to 40 percent of males with SCD experience priapism, usually starting in adolescence. The pathophysiology is linked to nitric oxide depletion, dysregulation of phosphodiesterase type 5, oxidative stress, and genetic factors. Although common, priapism is often overlooked and undertreated, which can lead to erectile dysfunction and psychological problems. A 21-year-old African American man with no prior medical history presented to Henry Ford St. John Hospital with a painful erection of four days' duration. Laboratory evaluation revealed hemoglobin of 11.5 g/dL (reference range: 13.5-17.5 g/dL) and a positive sickle cell solubility test. Hemoglobin electrophoresis confirmed SCD (HbS 61.9%, HbF 35.9%). Initial interventions, including cavernosal aspiration and phenylephrine injection, were unsuccessful, necessitating distal cavernosal shunt surgery. Hydroxyurea therapy was initiated, and the patient was discharged. Four days later, the patient presented a recurrence of priapism. Symptoms resolved after red blood cell exchange transfusion. He was discharged on hydroxyurea and sildenafil, with multidisciplinary follow-up arranged. This case shows that patients without a prior SCD diagnosis can present with priapism as the first sign, even without typical lab abnormalities. Early recognition and prompt treatment, including urologic procedures, hydroxyurea, and red blood cell exchange transfusion, are important to prevent recurrence and long-term complications. Collaboration among urology, hematology, and mental health specialists is crucial for the care of this patient population.

## Introduction

Priapism is an erection lasting longer than four hours, unrelated to sexual stimulation, and is considered a urologic emergency [1]. Stuttering priapism is a recurrent subtype of acute ischemic priapism characterized by repeated episodes of painful, unwanted erections separated by periods of detumescence [2]. Prompt recognition and management are essential to prevent long‑term complications such as erectile dysfunction and psychological distress [3].

Ischemic priapism accounts for approximately 95% of cases and is most associated with hematologic disorders, particularly sickle cell disease (SCD) [4]. SCD is an inherited hemoglobinopathy characterized by chronic hemolysis, vaso‑occlusion, and progressive organ damage [5].

Globally, SCD affects approximately 7.74 million people, with an estimated 515,000 births affected annually. The highest disease burden occurs in sub‑Saharan Africa, where up to 2% of newborns are affected [6]. In the United States, SCD occurs in approximately 1 in 365 African American and 1 in 16,300 Hispanic American births [7]. Newborn screening programs expanded throughout the late twentieth century and became universal by 2006, significantly improving early diagnosis and reducing SCD‑related morbidity and mortality [8,9].

Priapism is a frequent complication in males with SCD, typically presenting during adolescence or early adulthood [4]. It may manifest as recurrent brief episodes (stuttering priapism) or as prolonged ischemic events requiring emergency intervention [2,3]. The pathophysiology of SCD‑related priapism is multifactorial, involving nitric oxide depletion from chronic hemolysis, phosphodiesterase‑5 dysregulation, oxidative stress, heme toxicity, and endothelial dysfunction [4,5,10]. Although genetic susceptibility has been explored, particularly polymorphisms in NOS3 and EDN1, large genetic association studies have demonstrated no significant differences between patients with and without priapism [11].

Despite its prevalence, priapism in SCD remains frequently underrecognized and undertreated. Erectile dysfunction affects a substantial proportion of men with recurrent ischemic priapism, and men with SCD have more than twice the rate of erectile dysfunction compared with those without SCD [12,13]. These clinical complications are accompanied by significant psychological distress [14].

## Case presentation

A 21-year-old African American male with no known medical history came to the emergency department of Henry Ford St. John Hospital after four days of painful, persistent penile erection. He had severe pain and trouble urinating. He reported prior episodes that resolved on their own, but this one lasted longer and was more severe. The patient denied drug or medication use and had no personal or family history of anemia, sickle cell trait or disease, or other hematologic disorders. Physical examination revealed a fully rigid and tender penis, consistent with ischemic priapism. Laboratory evaluation showed a hemoglobin level of 11.5 g/dL (reference range: 13.5-17.5 g/dL), and a sickle solubility test was positive. All other routine laboratory results were within normal limits.

**Figure 1:**
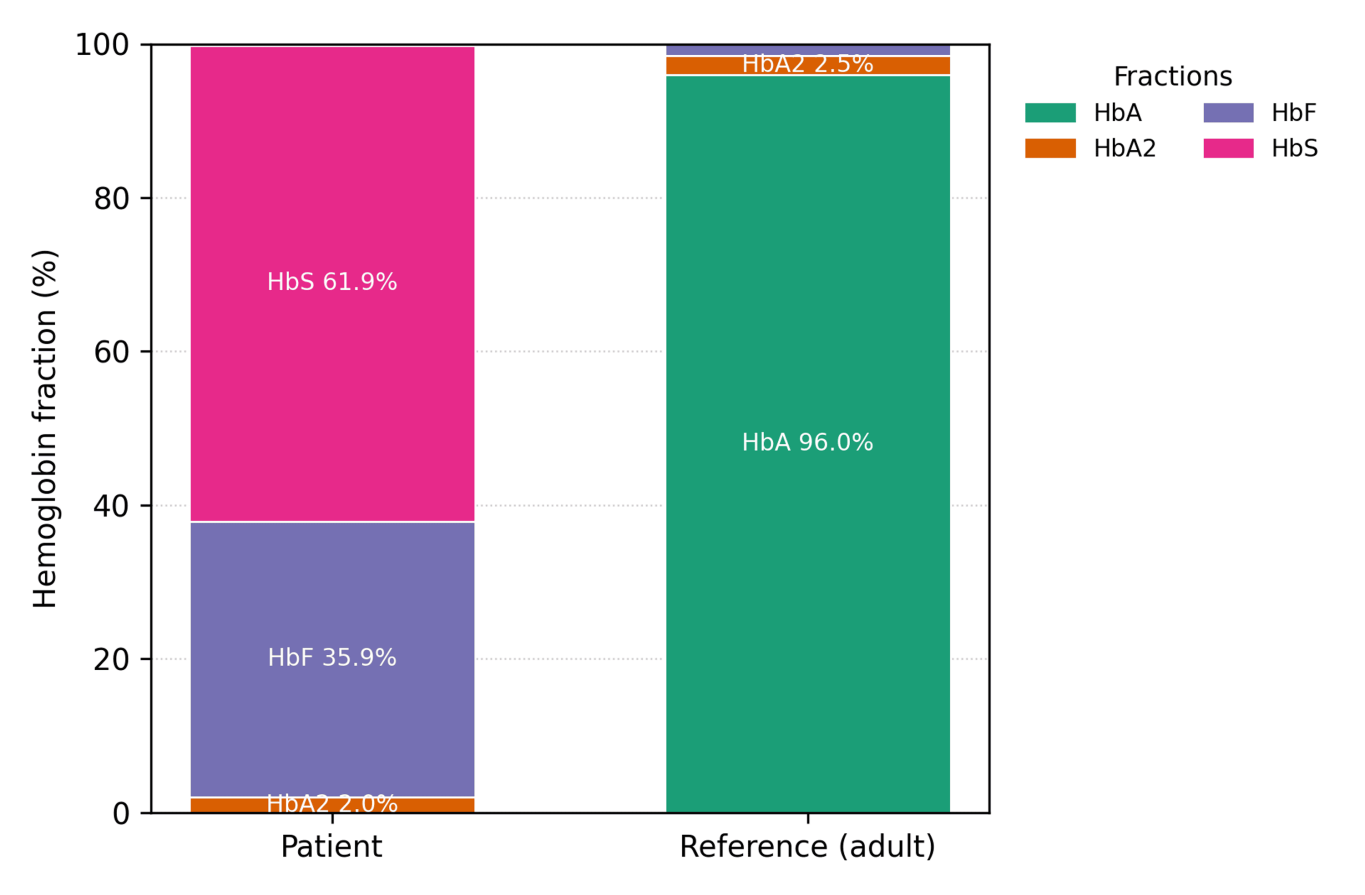
Hemoglobin electrophoresis results represented as stacked bar charts for the patient compared with adult reference values

Several emergency interventions were attempted, beginning with a penile nerve block using 6 mL of 2% lidocaine, followed by aspiration of cavernosal blood, yielding approximately 5 mL with only transient softening of the penile shaft before rigidity recurred. Intracavernosal phenylephrine was then administered, with two sequential injections of 100 µg into the corpus cavernosum, without successful detumescence. After consulting urology, the patient underwent distal cavernosal shunt surgery (Winter shunt), which resolved the priapism. Hemoglobin electrophoresis during hospitalization revealed 61.9% hemoglobin S and 35.9% hemoglobin F, with no detectable hemoglobin A (Figure 1). Although no absolute HbS cutoff exists, the predominance of HbS in the absence of HbA confirms the diagnosis of sickle cell disease. The hematology team was consulted, and oral hydroxyurea 500 mg twice daily was started. The patient was discharged with follow-up appointments for primary care, hematology, and urology. However, four days later, the patient returned with recurrent priapism. Cavernosal aspiration in the emergency department was unsuccessful. Hematology was consulted, and a red blood cell exchange transfusion was performed, which resolved the symptoms. After discussing with urology, sildenafil was started to help prevent recurrence. The patient was discharged again on hydroxyurea 500 mg orally two times per day and sildenafil 50 mg orally once daily, with counseling on medication adherence and symptom monitoring, and further follow-up was arranged.

## Discussion

This case illustrates the diagnostic and management challenges of priapism in a patient without a prior diagnosis of sickle cell disease and highlights the importance of hemoglobinopathy screening in such presentations.

The patient’s mild anemia and markedly elevated fetal hemoglobin (HbF 35.9%) likely masked classic features of SCD, delaying diagnosis. Elevated HbF is a well‑established disease modifier that reduces hemoglobin S polymerization and clinical severity, potentially obscuring diagnostic suspicion [5]. Universal newborn screening has improved early detection; however, adults born before widespread implementation or outside the United States remain at risk for delayed diagnosis [8,9].

Early identification of SCD enables initiation of disease‑modifying therapy, most notably hydroxyurea, which increases HbF, decreases hemolysis, and reduces vaso‑occlusive complications [5]. In refractory priapism, red blood cell exchange transfusion is effective for rapidly lowering hemoglobin S levels and preventing early recurrence [15]. Delayed intervention may result in penile fibrosis and permanent erectile dysfunction [1].

Beyond physical morbidity, priapism imposes a substantial psychosocial burden that adversely affects mental health, sexual function, physical well‑being, and quality of life. Feelings of shame, anxiety, depression, and delayed care‑seeking behavior are common. A multidisciplinary, patient‑centered approach incorporating routine screening with validated tools such as the PHQ‑9, International Index of Erectile Function, and Erection Hardness Score is essential [14,16].

Current AUA/SMSNA guidelines emphasize that acute ischemic priapism is a urologic emergency requiring immediate intervention. First‑line management includes corporal aspiration with or without irrigation and intracavernosal phenylephrine. Surgical intervention, preferably distal shunting, is recommended for refractory cases. In patients with SCD, red blood cell exchange transfusion should not delay standard urologic therapy, though chronic transfusion may be considered for secondary prevention [3].

For long‑term prevention, hydroxyurea and phosphodiesterase‑5 inhibitors, such as sildenafil or tadalafil, are commonly used. Systematic reviews suggest PDE‑5 inhibitors reduce recurrence without significant adverse effects [17]. Although high‑quality evidence supporting combination therapy remains limited, post‑hoc analysis of randomized trial data suggests potential benefit from combining hydroxyurea with tadalafil for the prevention of recurrent priapism [18]. With long‑term hydroxyurea and sildenafil therapy, this patient has remained free of recurrent priapism requiring hospitalization, underscoring the importance of combined pharmacologic therapy and multidisciplinary care between hematology, urology, and mental health specialists.

## Conclusions

Priapism may present as the first clinical manifestation of sickle cell disease in young adults, even without previous symptoms or family history, particularly among individuals born prior to universal newborn screening or born outside the United States. Therefore, screening for hemoglobinopathies is essential in patients with unexplained priapism. Early identification, prompt treatment, and coordinated multispecialty care are vital to reduce the risk of long‑term complications, including erectile dysfunction and mental health disorders.
